# Operationalising ethics in the EHDS: Tools, principles, and governance mechanisms

**DOI:** 10.12688/openreseurope.23447.1

**Published:** 2026-06-03

**Authors:** Kristina Weishäupl, Beatriz Jacinto Barros, Lisa Ferent, Anne Sofie Skødt Jørgensen, Alexander Leander Knudsen

**Affiliations:** 1 Digital Health and Data Infrastructures, Austrian National Public Health Institute, Vienna, 1010, Austria; 2 Innovation in Health Information Systems, Sciensano, Brussels, 1050, Belgium; 3 Danish Health Data Authority, Copenhagen S, 2300, Denmark

**Keywords:** TEHDAS2, Health Data, European Health Data Space, Health Data Access Body, Secondary Use, Data Ethics, Data Solidarity

## Abstract

The European Health Data Space (EHDS) offers significant opportunities for the secondary use of health data to support research, innovation, and public health across Europe. However, it introduces complex ethical governance challenges. While the EHDS Regulation requires data access applications to address ethical considerations, it does not establish a uniform EU-level ethics review pathway, leaving oversight largely to national law and institutional competence. This variability risks fragmentation, procedural inconsistency, and barriers to cross-border data access.

This article presents recommendations for operationalising ethical governance under the EHDS, informed by an EU-wide, multi-stakeholder workshop conducted under the TEHDAS2 Joint Action. Insights were analysed thematically in relation to the EHDS user journey and relevant governance frameworks, yielding recommendations across three interconnected areas: First, harmonisation with national flexibility calls for shared minimum ethical standards and a voluntary common ethics reference framework to support consistent assessments without constraining national autonomy. Standardised documentation and mutual recognition agreements can further reduce duplication while facilitating cross-border secondary use. Second, building public trust, security, and transparency require making ethical governance visible and intelligible through accessible summaries of approved data uses, clear communication on data security safeguards, and inclusive citizen engagement. Healthcare professionals are identified as key trusted intermediaries whose informed involvement can reinforce confidence in the system. Third, building capacity and expertise for ethical assessment emphasises the need for interdisciplinary skills spanning bioethics, artificial intelligence ethics, data protection, and health informatics within Health Data Access Bodies and ethics committees. EU-supported knowledge exchange, mentorship schemes, and shared digital platforms can help mitigate disparities across Member States.

Together, these recommendations offer a practical roadmap for policymakers, Health Data Access Bodies, and stakeholders to establish ethical governance that is coherent, transparent, and oriented toward the collective public good as the EHDS becomes operational.

## Plain Language Summary

The European Health Data Space (EHDS) is a new EU law that aims to make health data easier to reuse for the benefit of people across Europe. Reuse of health data means using data for purposes other than the one it was originally collected for, such as research, innovation, or public health planning. This can help improve healthcare, develop new treatments, and support better policy decisions. At the same time, using health data in this way raises important ethical questions about privacy, fairness, trust, and accountability.

Under the EHDS, organisations must explain how they will handle ethical issues when applying to reuse health data. However, there is currently no common European process for ethical review. Instead, each country can decide for itself whether and how to assess ethical aspects. The risk of different approaches can lead to confusion, uneven protection, duplicated work, and difficulties when data are reused across borders. The way these issues are addressed is often called “ethical governance”.

This article shows how ethical governance under the EHDS could work in practice. The recommendations are based on discussions from an EU-wide workshop with many stakeholders under the TEHDAS2 Joint Action.

Three main needs were identified:

• First, better alignment across Europe is needed, for example through shared minimum ethical standards and mutual recognition of ethical reviews, while still respecting national differences.
• Second, ethical governance should be transparent and easy for citizens to understand, to help build trust in how health data are used.
• Third, it needs stronger skills and expertise for ethical reviews, especially in areas like data protection, health informatics, and artificial intelligence.

Together, these proposals offer practical guidance for putting ethical governance into practice as the EHDS becomes reality.

## Introduction

The European Health Data Space (EHDS) Regulation ^1^ offers significant opportunities across Europe for the secondary use of health data, which is defined as the reuse of health data for purposes such as research, innovation, public health, policy-making, and personalised medicine. By enabling broader and more systematic reuse of health data, the EHDS has the potential to accelerate knowledge generation and improve healthcare and the health of the European population. At the same time, it raises important ethical considerations. The EHDS Regulation, notably Article 67(2)(j), requires data access applications to address ethical considerations, and allows existing national ethics assessments to substitute applicant’s own ethical statements where mandated. However, the EHDS does not establish a uniform EU-level ethics review pathway, thus remaining unclear where and how ethical aspects should be incorporated into the overall health data access application assessment process, conducted by Health Data Access Bodies (HDABs). Ethical oversight remains primarily governed by national law and institutional competence, leaving it up to the Member States to define how ethical review interacts with HDABs.

Implementing ethical governance under the EHDS faces multiple challenges, both at the level of implementation and in day-to-day operation. At the implementation level, national ethics procedures remain fragmented, and the absence of harmonised standards or mutual recognition can create bottlenecks and slow the development of coherent cross-border approaches. The role of HDABs in ethical oversight is often unclear, particularly where they may simultaneously act as data holders or evaluators, highlighting the need for clear governance boundaries to preserve independence and transparency. Additionally, Member States or institutions might not have sufficient capacity, expertise, or resources to conduct timely and rigorous ethical assessments, especially for complex, cross-border, or novel use cases. At the operational level, the absence of harmonised standards introduces variability and procedural complexity that can hinder cross-border secondary use and create uncertainty for data users. Public trust depends on visible, intelligible, and accountable ethical governance. Without transparent communication on data security, safeguards, and ethical processes, citizens and healthcare professionals may resist participation or disengage from the system. Achieving a trustworthy and effective ethical framework therefore requires coordinated action across Member States. While ethical guidance will remain a national competence, strategic alignment is essential to ensure coherent progress, mutual understanding, and consistent standards that safeguard public trust.

This article presents recommendations to support EHDS implementation, informed by an EU-wide, multi-stakeholder workshop held under the TEHDAS2 Joint Action. ^2^ The workshop brought together participants including policymakers, HDAB representatives, ethics committee members, data protection experts, researchers, and patient and civil society representatives, providing a cross-sectoral perspective on the ethical governance challenges ahead. The workshop aimed at an exchange of views and identification of shared priorities, and its insights were analysed thematically, taking into account the EHDS user journey and relevant frameworks for ethical governance. ^3^ These form the basis for recommendations covering infrastructure, processes, and the operationalisation of ethical oversight across three interrelated areas: 1) harmonisation with national flexibility, 2) public trust, security, and transparency and 3) building capacity and expertise for ethical assessment. Together, they aim to support policymakers, HDABs, and ethics committees, data protection authorities, researchers, and patient representatives in creating an ethical governance framework that is coherent, practical, and oriented toward the collective public good, ensuring that the EHDS can deliver its full potential in a secure, transparent, and socially responsible manner.

## Recommendations

The following sections present targeted recommendations to support ethical governance of secondary use under the EHDS. Each topic addresses key dimensions of ethics, and within each of them, practical recommendations are provided for EU institutions, Member States, and HDABs, offering guidance on actionable steps to strengthen transparency, consistency, and societal benefit while respecting national responsibilities and diverse contexts. The key topics and associated recommendations are summarized in Table 1.

**Table 1.** Topics and key recommendations.

Topic	Key recommendations
Harmonisation with national flexibility	• Establish shared minimum ethical standards • Create a voluntary EU “common ethics reference framework” • Harmonise documentation and transparency, not national mandates • Embed cross-country coordination and mutual recognition into EHDS governance o Data solidarity framework as an illustrative example in Table 2
Public trust, security, and transparency	• Make ethical governance visible, intelligible and embedded in EHDS structures • Enhance communication on data security and confidentiality • Facilitate informed and inclusive citizen engagement • Leverage healthcare professionals as trusted intermediaries
Build capacity and expertise for ethical assessment	• Clarify role of HDABs in ethical considerations • Provide information, guidance and transparency on ethical requirements • Develop interdisciplinary expertise and organizational capacity • Facilitate EU-supported knowledge exchange and cross-country collaboration

### Harmonisation with national flexibility

Ethical considerations are central to the secondary use of health data under the EHDS, yet the Regulation does not provide detailed guidance on what such considerations entail. If further national specifications are absent, stakeholders, including HDABs and data users, must interpret principles such as public interest and societal benefit themselves, which risks inconsistency and uncertainty. Effective ethical governance for the secondary use of health data under the EHDS requires a careful balance between convergence and national diversity. While ethics assessment remains a national competence, divergent practices across Member States risk fragmentation, legal uncertainty, and unnecessary administrative burden, potentially slowing access and constraining the public health and societal value that the EHDS is designed to deliver. At the same time, ethical review is deeply embedded in national legal frameworks, healthcare systems, and societal expectations, making fully harmonised EU-level procedures neither proportionate nor feasible. Harmonisation should therefore focus on structured alignment (e.g. convergence around shared principles, minimum expectations, and transparent practices) while preserving flexibility in interpretation, institutional design, and decision-making. A foundational element of this approach is the articulation of shared minimum ethical standards. These standards would not dictate assessment decisions but define core ethical dimensions that all assessments of data applications are expected to consider. They operationalise widely recognised values such as public interest, societal benefit, proportionality, and solidarity, into concrete points of attention, guiding evaluation of how proposed uses contribute to public health objectives, balance expected benefits against risks, and implement safeguards such as data minimisation and protection against re-identification. Minimum standards would also encourage systematic consideration of fairness, inclusion, impacts on vulnerable groups, and the protection of professional ethics, medical confidentiality, and trust in clinical relationships. Framed as a common reference rather than an additional procedural layer, these standards could be integrated into existing national review processes and the work of HDABs without disrupting established practices. Critically, these standards should embed a proportionality and risk-based approach: the depth and intensity of ethical review should be calibrated to the actual risk and context of use, rather than applied uniformly across all data types. Given the diversity of data involved in EHDS secondary use (including omics, imaging, electronic health records, registries, digital apps outputs, among other 17 categories of data), a differentiated assessment approach is both necessary and proportionate. It is worth noting that the principled foundation for such assessments is already well established through instruments such as the Declaration of Helsinki, the Taipei Declaration on Ethical Considerations Regarding Health Databases and Biobanks, and the Oviedo Convention. In practice, many bottlenecks arise not from an absence of ethical principles but from heterogeneous interpretations of shared standards across ethics committees. Minimum standards, framed as interpretive guidance rather than new rules, can help address this by anchoring divergent national readings to a common reference.

Building on these standards, a voluntary EU-level “common ethics reference framework” could provide practical support for nationally led assessments. By adopting a code-of-conduct-style mechanism rather than a static checklist, the framework would be consistent across Europe while remaining modular, technology-sensitive, and adaptable to national contexts, with provision for periodic revision as data uses, technologies, and societal expectations evolve. The framework could include standard templates for ethics sections of data access applications, structured guiding questions addressing public interest, risk–benefit balance, safeguards, as well as decision-support tools to enhance transparency and reproducibility. Curated case studies covering diverse data types and use cases such as algorithm training, registry linkage, or insurance data, could illustrate how comparable ethical questions have been addressed in different national contexts, making variation more legible for applicants, reviewers, and the public. The voluntary and modular nature of the framework ensures alignment and mutual learning without constraining national autonomy. Developing common ethical frameworks across Member States can further support consistency and transparency by helping to guide data applicants, supporting ethical committees, and informing the public about ethical considerations. Importantly, frameworks should be co-created with diverse stakeholders, including citizens and data subjects, to ensure that definitions of public interest and societal benefit reflect shared values. While comprehensive frameworks can increase procedural complexity, they also provide clarity and guidance, particularly for data users with limited prior experience in ethical assessment. Ultimately, the effectiveness of any common framework depends not only on its design but on the degree of trust and mutual recognition between ethics committees across Member States. Without a shared understanding that assessments conducted under comparable standards in one jurisdiction can be relied upon in another, even well-designed frameworks risk producing parallel, duplicative reviews that defeat the purpose of harmonisation. Strengthening mutual recognition is therefore not a secondary consideration but a precondition for the framework to deliver its intended value. An example of how such a framework can be operationalised is provided in Table 2.

**Table 2.** Example: Data Solidarity Framework.

An example of a framework suited to consider - among other things - the ethical aspects of secondary use is the data solidarity framework. ^4^ This framework moves beyond abstract principles such as fairness, accountability, and inclusion by operationalising them in terms of how the benefits and burdens of data use are distributed across populations. It foregrounds the importance of equitable risk–benefit sharing and prioritises outcomes that generate meaningful public value, particularly in the context of public health. The three pillars of data solidarity - facilitating data use that creates public value, preventing and mitigating harms, and ensuring that commercial benefits are shared with communities - offer a coherent normative and practical foundation for the EHDS. Embedding these pillars across EHDS policies, instruments, and evaluation practices would help ensure that data governance does not merely enable access and innovation but actively contributes to socially just outcomes. Concretely, the framework could be integrated into standardised assessment procedures and operational checklists. This would provide actionable guidance for policymakers and practitioners to systematically consider questions of harm mitigation, collective benefit, and fair value distribution, thereby strengthening both the ethical robustness and societal legitimacy of EHDS initiatives.

Convergence can also be advanced by harmonising documentation and transparency. Standardised documentation, including structured summaries of ethics opinions, public interest justifications, safeguards applied, and, where relevant, stakeholder involvement, can make assessments more comparable and interpretable across jurisdictions. A shared taxonomy of ethical risks, benefits, and safeguards can further support cross-border projects, clarifying when an ethics assessment from one Member State may be relied upon and when additional, context-specific review is warranted. Bilateral or multilateral agreements on mutual recognition of ethical assessments, grounded in normative principles such as data solidarity, can reduce duplication and improve efficiency. Such agreements should be understood as non-binding coordination tools that complement rather than override national legal frameworks, as their application would be contingent on conditions such as alignment with shared minimum standards, comparable data sensitivity classification, and equivalent safeguard requirements - criteria that EHDS governance bodies should develop as implementation progresses. By reducing duplication while preserving national decision-making authority, harmonised documentation facilitates multi-country secondary use without imposing uniform outcomes. Importantly, sustainable alignment requires embedding cross-country coordination within EHDS governance structures. The specific mechanisms to achieve this, including networks of assessors, knowledge-sharing platforms, and targeted support for less-resourced Member States, are addressed in the capacity-building section below.

### Public trust, security, and transparency

Public trust constitutes a fundamental prerequisite for the effective operation of the EHDS. Without sustained confidence from citizens, patients, and healthcare professionals, even robust governance arrangements risk being perceived as illegitimate or intrusive, potentially leading to resistance, disengagement, or increased opt-out rates. Trust extends beyond legal compliance and technical safeguards to encompass the visibility, credibility, and intelligibility of ethical governance processes, alongside clear communication regarding the use, protection, and oversight of health data. The expansion of secondary use under the EHDS, including a broad range of data categories, a wider set of actors and new bodies, and cross-border applications, may amplify concerns related to confidentiality, potential misuse, and loss of control over personal health information. At the same time, disparities in digital maturity and governance capacity across Member States may lead to uneven perceptions of security and fairness. In this context, trust cannot be assumed as an automatic outcome of regulation; it must be deliberately cultivated through transparency, inclusive stakeholder engagement, and demonstrable safeguards, embedded within EHDS governance. Trust is not merely a matter of communication: it is also shaped by whether governance functions effectively in practice. Weak or inconsistent ethical assessments will erode confidence regardless of how well they are communicated. The recommendations in this section should therefore be understood as interdependent with the harmonisation and capacity-building measures set out in the preceding and following sections.

One critical pathway for fostering trust lies in making ethical governance visible and understandable. Ethical review processes, which play a central role in safeguarding societal values, often operate with limited public visibility. Increasing transparency regarding how ethical considerations are addressed, through publicly accessible summaries or dashboards of approved data uses, can demonstrate accountability, and show that ethical assessment is substantive and reflective rather than procedural. Examples of such platforms are already deployed, such as the EU Clinical Trials Register, ^5^ which provide publicly accessible summaries of approved research. A comparable register for secondary health data use maintained at HealthData@EU level could make governance more visible to citizens and professionals across Member States. Importantly, these communications should articulate the public interest rationale, key ethical considerations, and applied safeguards, without disclosing sensitive or proprietary information, thereby strengthening confidence in governance mechanisms.

Targeted communication on data security and confidentiality is essential to reinforce trust. While the EHDS establishes robust legal and technical safeguards, their credibility depends on clear articulation to both data subjects and healthcare professionals. This includes explaining the use of Secure Processing Environments, access control and monitoring mechanisms, and practical measures to mitigate re-identification risks. Particular attention should be paid to differences in digital capacity and governance maturity across Member States, as perceptions of weaker protection in certain jurisdictions could undermine confidence in cross-border secondary use. Clear, consistent messaging on minimum security standards and oversight mechanisms helps ensure stakeholders perceive EHDS data use as uniformly protected and accountable.

Equally important is the facilitation of informed and inclusive citizen engagement. Trust is closely tied to understanding of data rights, including opt-out options, the purposes for which health data may be reused, and the collective and individual benefits of this reuse. Engagement strategies should be accessible, inclusive, and responsive to different levels of digital literacy, supported by EU-level tools, such as shared communication materials, explanatory modules, or interactive resources, that Member States can adapt locally. Beyond communication, participation mechanisms can further strengthen trust. These may include patient and citizen representation on ethics committees or HDABs, as well as citizen panels or public consultations. This type of involvement is where more durable trust is built and where meaningful operationalisation of “public interest” and “societal benefit” becomes possible. Special attention should be paid to digitally marginalised populations and to avoiding additional burdens for individuals facing barriers to healthcare access or digital participation. In the EHDS, opt-out is a rights-based mechanism designed to protect autonomy and choice, not a measure of public trust. Nonetheless, where significant variation across Member States is observed, this may usefully prompt reflection on whether communication and engagement strategies are reaching citizens effectively.

Healthcare professionals also play a pivotal role as trusted intermediaries. Their perceptions of confidentiality, professional responsibility, and administrative burden shape wider trust in the EHDS. Ensuring that professionals are well-informed about the operation of secondary use, ethical safeguards, and the respect for professional obligations reinforces confidence that secondary use supports rather than undermines clinical practice. In practice, this could include structured briefing programmes for clinicians on how patient data is used downstream, developed in collaboration with professional associations such as medical councils and nursing associations. Closing the loop by informing clinicians about research outputs generated from data collected in their practice, could reinforce the value of high-quality data entry and strengthen the link between primary care and secondary use.

Finally, these efforts should be integrated into EHDS governance rather than treated as ancillary activities. Embedding transparency, communication, and engagement into the operational routines of HDABs and HealthData@EU structures ensures sustained trust-building, supported by dedicated resources and expertise. Systematic monitoring of public perceptions and emerging concerns allows governance arrangements to adapt proactively, supporting the responsible secondary use of health data across Europe.

### Build capacity and expertise for ethical assessment

Effective EHDS governance depends on clarity about how HDAB procedures interact with ethical review arrangements under national law. Member States should clarify these interactions as part of their national implementation arrangements, ensuring appropriate coordination between HDABs and ethics bodies. Critically, ethics review should not be understood as a standalone safeguard or a single gate to be passed. It is one component of a broader compliance ecosystem that institutions must be able to deliver, combining data protection, scientific validity, public-interest justification, patient safeguards, and transparency. An ethics committee provides a valuable perspective, but it is not the only consideration that ultimately leads to a data access permit being issued. Importantly, transparency alone does not make a practice lawful, and legality and ethics may at times be in tension, requiring overarching institutional decision-making that integrates ethics but is not limited to it. The quality of ethical governance therefore depends on institutions building mechanisms that are robust, sustainable, reliable, self-correcting, and adaptable over time, where trust is understood not as a one-time approval but as a continuous dialogue between institutions and the communities they serve.

The effective and timely evaluation of ethical considerations in the secondary use of health data under the EHDS depends on adequately trained personnel, sufficient resources, and access to interdisciplinary expertise. Ethical assessment increasingly extends beyond traditional bioethical review. It now encompasses technical and domain-specific knowledge, including data protection, health informatics, artificial intelligence, legal frameworks, cross-border data flows, and emerging analytical methodologies such as AI. Enhancing expertise in these areas enables assessors to evaluate potential benefits, risks, and societal implications with nuance and confidence. Similarly, the growing volume and complexity of secondary use requires a broader, flexible, and continuously updated skill set. Without sufficient capacity, both HDABs and ethics committees risk bottlenecks, inconsistent assessments, or overly procedural approaches that fail to capture the societal, clinical, and technological implications of secondary use. Equally important are investments in recruitment, structured training, professional development, and organisational infrastructure, including standard operating procedures, assessment frameworks, and ethical guidance. Career pathways and incentives for participation in secondary use oversight can help attract and retain skilled personnel, while practical, case-based training ensures that expertise remains up to date with evolving EHDS-specific challenges. Disparities in staffing levels and expertise across Member States may result in unequal implementation, potentially delaying access and undermining the credibility and efficiency of EHDS governance. A cornerstone of addressing these challenges lies in developing both interdisciplinary expertise and robust organisational capacity. This includes not only ethics expertise but also fluency in data protection law, scientific methodology, and public-interest assessment, reflecting the integrated nature of the compliance ecosystem HDABs are expected to navigate.

Finally, capacity could be further strengthened through EU-supported knowledge exchange and cross-country collaboration. An important consideration for these coordination efforts is that HDABs are still in the process of being built, staffed, and tested across Member States. While meaningful engagement in harmonisation or cross-country knowledge exchange will only be possible once these institutions are fully operational and functioning reliably, the requirements for such coordination need to be considered already at the design and planning stage. Doing so will help ensure that, once operational, HDABs are able to participate effectively in these activities without requiring substantial restructuring. Insights on the importance of exchange can be gleaned from another European Regulation: The Clinical Trials Regulation (CTR). Although the CTR aimed to harmonise the assessment and supervision of clinical trials by introducing a single EU portal and common procedures, the ethics component of clinical trial assessment remains at Member State level, as it lies outside the EU’s direct competence. Sustained exchange among ethics committees at both national and European level has supported gradual convergence, but this has required continuous effort rather than occurring automatically in response to the Regulation. This precedent suggests that convergence among ethics bodies across the EHDS should not be assumed to emerge spontaneously, it will require sustained political will and active engagement from citizens, patient groups, researchers, and professional communities. With that foundation in mind, the following mechanisms can support gradual and meaningful alignment. Mapping existing ethics bodies and networks and engaging them in the development of shared principles would enable knowledge exchange and gradual convergence while respecting national legal and ethical contexts. Initiatives such as networks of ethics assessors, joint workshops, mentorship schemes, and shared digital platforms for case studies and standardised tools allow Member States to benefit from collective expertise while fostering mutual learning and alignment. Such mechanisms are particularly important for less-resourced systems, helping to mitigate disparities and ensuring that all countries can conduct timely, high-quality ethical assessments. Ensuring meaningful participation from all Member States may require targeted support, shared resources, and training opportunities. Systematic monitoring of capacity, including assessment turnaround times, training uptake, and projected caseloads, can inform iterative improvements and ensure national capabilities evolve alongside EHDS-wide needs.

Equally important is providing guidance and transparency on ethical requirements. While Member States are responsible for defining national ethics obligations, HDABs should facilitate access to this information and support applicants throughout the process. Structured procedures should enable feedback loops between data holders, users, and the public, ensuring that ethical considerations are actively documented and continuously refined. Transparent communication strengthens accountability, promotes informed assessments, and supports responsible data stewardship.

By embedding interdisciplinary training, organisational support, and cross-country collaboration within EHDS governance, Member States can collectively strengthen the quality, consistency, and timeliness of ethical review, reinforcing both public trust and the realisation of societal and public health benefits across Europe.

## Conclusion

This article illustrates how ethical governance of secondary use under the EHDS requires coordinated action across multiple levels, combining EU guidance, national structures, and the operational role of HDABs. The recommendations presented provide a practical roadmap for immediate steps, including clarifying responsibilities, strengthening expertise, promoting transparency, and fostering trust among citizens, professionals, and other stakeholders.

While these measures lay the foundations for ethical oversight, several priority questions warrant sustained attention as implementation progresses. How can emerging technologies such as artificial intelligence be evaluated ethically across diverse legal and cultural contexts? What constitutes meaningful involvement of citizens and patients in shaping ethical frameworks, and how can public value be operationalised in practice? How can ethics processes remain dynamic and responsive as secondary use evolves, without creating procedural bottlenecks? Addressing these questions will require sustained investment, dialogue, and experimentation, combining technical guidance with participatory governance. By embedding ethical reflection into the design, operation, and oversight of the EHDS, stakeholders can ensure that secondary use not only complies with legal and procedural requirements but also upholds societal trust, maximizes public benefit, and supports responsible innovation in health research and policy.

### Ethics and consent

Ethical approval and consent were not required.

## Disclaimer

The views expressed in this article are those of the author(s). Publication in Open Research Europe does not imply endorsement of the European Commission.

## Data Availability

The data for this article consists of bibliographic references, which are included in the References section.
