# Supplementary material for: Operationalising ethics in the EHDS: Tools, principles, and governance mechanisms
Source: Open Res Eur. 2026 Jun 3;6:191. [Version 1] doi: 10.12688/openreseurope.23447.1 (PMC13560822; doi:10.12688/openreseurope.23447.1)
Supplement: Peer review history

# Reviewer report: Reviewer response for version 1

**DOI:** 10.21956/openreseurope.25373.r78031

**Published:** 2026-08-12

**Authors:** Jorge M. Mendes, Lúcia Domingues

**Affiliations:** 1 NOVA Medical School | Faculdade de Ciências Médicas, Universidade Nova de Lisboa Comprehensive Health Research Centre (Ringgold ID: 587327), Lisbon, Lisbon, Portugal

**General appreciation**

 We read this Open Letter with real interest and a good deal of agreement. It addresses one of the most pressing gaps in the current EHDS landscape: the Regulation opens the door to large-scale secondary use of health data but leaves the ethical governance of that use largely to national competence, without a common review pathway. We also read it for what it is — the position of a consortium, informed by a multi-stakeholder workshop convened under the TEHDAS2 Joint Action — and at a moment when Health Data Access Bodies are still being built, a piece that tries to make ethical governance concrete and actionable is genuinely valuable. As an Open Letter is meant to, it raises awareness, frames the problem well, and points in a constructive direction. We see this as a priority conversation, and we would like our comments read in that spirit: as help to make a worthwhile letter as strong as it can be.

 This open letter addresses a timely and consequential aspect of the implementation of the European Health Data Space. The manuscript is clearly written, logically organised and accessible to both specialist and policy audiences. Its division into harmonisation, public trust and capacity-building provides a useful structure, while Tables 1 and 2 help summarise the principal recommendations. The emphasis on proportionality, transparency, interdisciplinary competence and cross-country coordination is well justified.

 However, the manuscript presents its recommendations as derived from an EU-wide multi-stakeholder workshop and a thematic analysis, without providing sufficient methodological details to assess how those recommendations were developed. While the policy arguments are persuasive on their own terms, the path from the workshop to the recommendations is not yet visible to the reader. We want to be explicit about how we read the piece: as an Open Letter reporting the sense of a workshop held under the TEHDAS2 Joint Action, not as an empirical study, and, consistent with that format, we are not asking the authors to add new data and analysis or to turn it into a qualitative study. But precisely because these are recommendations, a proportionate account of how they were derived from the workshop is fundamental rather than optional. Since no separate publication appears to document the workshop, that short account needs to live here.

**Specific comments**

**Clarify the nature of the manuscript.**

 The article combines features of a normative policy paper, an expert consensus exercise and a qualitative workshop study. The authors should clarify which of these is its principal methodological basis. This distinction matters because the manuscript repeatedly states that workshop insights were “analysed thematically”, which implies a systematic empirical analysis rather than an informal consultation.

 If the workshop was primarily consultative, the manuscript should describe the recommendations as expert-informed proposals. If it were treated as a qualitative study, considerably more methodological reporting would be required.

**Give a short, proportionate account of how the workshop informed the recommendations.**

 The methodological information currently provided in the Introduction does not yet let readers see how the recommendations were reached, which for a set of recommendations is fundamental. This need not be a methods section in the empirical sense; even briefly, and proportionate to an Open Letter, it would help to know:

- the date, duration and format of the workshop;
- the number of participants and countries represented;
- the distribution across stakeholder groups;
- how the workshop outputs were subsequently consolidated

**Separate workshop findings from authors’ policy interpretation**

 The current recommendations are presented as a continuous narrative, making it difficult to determine which statements emerged directly from participants, which originated from existing frameworks and which reflect the authors’ subsequent interpretation. We raise this proportionately: even a short paragraph, or a sentence, distinguishing what emerged from the workshop from the authors’ own policy reading would meet the point.

**Report divergent perspectives.**

 The manuscript acknowledges national differences but generally presents the recommendations as consensual. Ethical governance of secondary health-data use is likely to involve genuine tensions, including harmonisation versus national autonomy; transparency versus confidentiality and legitimate commercial interests; rapid access versus extensive ethical review; collective public value versus individual control; mutual recognition versus context-specific assessment; and standardisation versus flexibility.

 Reporting where participants disagreed would strengthen the paper rather than weaken it. It would also help identify which recommendations are mature enough for implementation and which remain proposals requiring further deliberation. Relatedly, and given the TEHDAS2 setting, we would gently caution against presenting the workshop as an EU-wide consensus; a brief note on which perspectives were more or less present would let readers weigh the recommendations accordingly.

**Explain the choice of the data-solidarity framework (Table 2).**

 Table 2 presents the data solidarity framework as an illustrative model. The rationale for selecting this framework should be explained. It is unclear whether it emerged prominently during the workshop, was selected by the authors after the workshop, or was chosen because of its conceptual compatibility with the recommendations. Alternative frameworks considered should also be briefly acknowledged.

**Improve the operational character of the recommendations.**

 The title promises tools, principles and governance mechanisms, but several recommendations remain at a relatively high level. Their practical value would be enhanced by specifying the institution responsible for each action; whether implementation should occur at the EU, national, or HDAB level; immediate, medium-term, and longer-term actions; dependencies between recommendations; possible indicators of implementation; and foreseeable resource requirements.

 For example, the proposed common ethics reference framework could be described in terms of its minimum components, governance arrangements, revision process and intended users. Similarly, mutual recognition would benefit from clearer eligibility conditions, exceptions and escalation mechanisms.

**Distinguish the layers of governance, and protect the independence of HDABs.**

 One addition that would strengthen the letter, and that sits close to our own area, is a clearer separation of the different things now gathered under “ethical governance”: ethical review, data-protection (GDPR) duties, the EHDS’s own statutory requirements such as the secure processing environment and the HDAB permit, and scientific validity. These engage different bodies and different legal bases, and the letter already senses this where it notes that legality and ethics can be in tension (Build capacity). Making the distinction a little more explicit would help readers see who decides what. In the same vein, the authors rightly observe that HDABs may act as both data holders and evaluators (Introduction); because that dual role is such a central ethical risk, we would welcome a few more words on how the independence of the ethical assessment is protected and how conflicts of interest at that level would be handled.

**Acknowledge the legal limits of mutual recognition.**

 The proposals for mutual recognition and for bilateral or multilateral agreements on ethics assessments (Harmonisation with national flexibility; Table 1) are attractive, and we support the direction. It would strengthen them to acknowledge, even briefly, that ethical review remains a national and often institutional competence that the Regulation does not itself harmonise. A short reflection on the legal footing for such recognition, on what basis a determination in one Member State could be relied upon in another, and what minimum equivalence would make that lawful, would keep the recommendation from reading as more settled than the current legal framework allows.

**A few of the substantive recommendations could be refined.**

 Three smaller points, offered constructively. First, the welcome proposal to publish public summaries or dashboards of approved data uses (Public trust, security and transparency) could note the safeguards (aggregation and minimum-cell thresholds) that keep a transparency register from itself disclosing sensitive information or enabling re-identification in small cohorts. Second, the characterisation of opt-out as “a rights-based mechanism… not a measure of public trust” (Public trust, security and transparency) is defensible but carries a normative choice; acknowledging that opt-out and trust interact would read as more even-handed. Third, where the letter discusses AI and algorithm training, a brief nod to the EU framework for artificial intelligence that will co-apply would help, as would a line on oversight that continues after the permit is granted, consistent with the letter’s desenvolva já conteúdo substantivo nas secções onde a proposta dá material (competency framework TRL/SRL, clinical immersion, tracks A/B)own image of trust as a continuous dialogue rather than a one-time approval (Build capacity).

**Avoid unsupported causal statements.**

 Several statements imply causal relationships, for example that weak ethical assessments will erode confidence, that limited communication may increase opt-out rates, or that informing healthcare professionals about downstream research outputs will improve data quality. These propositions are plausible, but the workshop design described in the manuscript cannot establish them.

 Such statements should either be supported with appropriate literature or expressed more cautiously as hypotheses, expectations or policy assumptions.

**Expand the supporting literature.**

 The reference list is unusually limited for the breadth of the manuscript. Important claims concerning public trust, citizen engagement, variability in ethics review, organisational capacity, mutual recognition, and the experiences of clinical-trial governance require wider support.

 The authors should distinguish between conclusions derived from the workshop and claims based on existing research or regulatory experience. References should also be added to support statements about national variation, institutional capacity and the role of healthcare professionals as trusted intermediaries.

**Moderate the conclusion.**

 The conclusion states that the recommendations provide a practical roadmap. Given the limited methodological reporting and absence of implementation testing, “proposed roadmap” or “initial framework for implementation” would be more appropriate. The recommendations should be understood as requiring validation through pilot implementation, stakeholder consultation and evaluation in different national contexts.

Where applicable, are recommendations and next steps explained clearly for others to follow? (Please consider whether others in the research community would be able to implement guidelines or recommendations and/or constructively engage in the debate)

Yes

Does the article adequately reference differing views and opinions?

Partly

Are all factual statements correct, and are statements and arguments made adequately supported by citations?

Partly

Is the rationale for the Open Letter provided in sufficient detail? (Please consider whether existing challenges in the field are outlined clearly and whether the purpose of the letter is explained)

Partly

Is the Open Letter written in accessible language? (Please consider whether all subject-specific terms, concepts and abbreviations are explained)

Yes

Reviewer Expertise:

Jorge M. Mendes (Data Science for Healthcare; Artificial Intelligence); Lúcia Domingues (Research Ethics; Clinical Tudies Operations)

We confirm that we have read this submission and believe that we have an appropriate level of expertise to confirm that it is of an acceptable scientific standard, however we have significant reservations, as outlined above.

# Reviewer report: Reviewer response for version 1

**DOI:** 10.21956/openreseurope.25373.r78035

**Published:** 2026-08-11

**Authors:** Lucia Mokra

**Affiliations:** 1 Comenius University in Bratislava, Bratislava, Slovakia

**Review:** Operationalising ethics in the EHDS: Tools, principles, and governance mechanisms

 1) Is the rationale for the Open Letter provided in sufficient detail? (Please consider whether existing challenges in the field are outlined clearly and whether the purpose of the letter is explained)

 The Open Letter discusses very relevant details connected with the implementation of the European Health Data Space Regulation. Beyond the regulation of the secondary health data, authors highlight the importance of ethical standards in the processing of health data, the lack of common standards – which may lead to discrepancies – and the potential benefits of a joint working version of a voluntary EU-level ‘common ethics reference framework’. As the most relevant argument for the rationale is put straight forwarded by the authors at the beginning: “Developing common ethical frameworks across Member States can further support consistency and transparency by helping to guide data applicants, supporting ethical committees, and informing the public about ethical considerations”.

 2) Does the article adequately reference differing views and opinions?

 Yes, the authors adequately reference different views in the topic, which is actually in the Centre of their Open letter – diversified ethical frameworks in different Member States.

 3) Are all factual statements correct, and are statements and arguments made adequately supported by citations?

 All factual statements are correct, corresponding to the research findings as well as the discussion in the multi-stakeholder workshop held under the TEHDAS2 Joint Action. Therefore, the majority of the findings as well as recommendations are concluded based on the discussion and overall data assessment.

 For the principle of differentiation assessment of health data collected based on EHDS, the reference to the other international instruments such as the Declaration of Helsinki, the Taipei Declaration on Ethical Considerations Regarding Health Databases and Biobanks, and the Oviedo Convention can be referenced more explicitly, by referencing concrete articles and the international treaty itself. Such an approach would complement the arguments provided and justified by authors.

 4) Is the Open Letter written in accessible language? (Please consider whether all subject-specific terms, concepts and abbreviations are explained)

 The Open Letter is written in a structured way, using both relevant linguistics and grammar, in an accessible way. All abbreviations, subject-specific terms and concepts are explained when first used and follow accordingly (e.g. The European Health Data Space (EHDS) Regulation or Health Data Access Bodies (HDABs).

 5) Where applicable, are recommendations and next steps explained clearly for others to follow? (Please consider whether others in the research community would be able to implement guidelines or recommendations and/or constructively engage in the debate)  

 The Open Letter focuses on developing recommendations for the processing of health data and on setting the framework for ethical standards in this regard.  The concluding part of the Open Letter argues for the need for clarity for Member States and their relevant health authorities and ethical authorities. There are concrete recommendations presented to support the building of capacity and expertise for ethical assessment, including concrete steps and suggestions. These reflect not only the recommendations themselves, but also suggestions for operationalisation, including competencies and financial support throughout the process.

Where applicable, are recommendations and next steps explained clearly for others to follow? (Please consider whether others in the research community would be able to implement guidelines or recommendations and/or constructively engage in the debate)

Yes

Does the article adequately reference differing views and opinions?

Yes

Are all factual statements correct, and are statements and arguments made adequately supported by citations?

Yes

Is the rationale for the Open Letter provided in sufficient detail? (Please consider whether existing challenges in the field are outlined clearly and whether the purpose of the letter is explained)

Yes

Is the Open Letter written in accessible language? (Please consider whether all subject-specific terms, concepts and abbreviations are explained)

Yes

Reviewer Expertise:

European law, European policies

I confirm that I have read this submission and believe that I have an appropriate level of expertise to confirm that it is of an acceptable scientific standard.

# Reviewer report: Reviewer response for version 1

**DOI:** 10.21956/openreseurope.25373.r75482

**Published:** 2026-07-15

**Authors:** Jens Declerck

**Affiliations:** 1 The European Institute for Innovation through Health Data, Oosterzele, Belgium

This paper addresses the operationalization of ethical governance within the European Health Data Space (EHDS), drawing on insights from a multi‑stakeholder workshop under the TEHDAS2 Joint Action. The manuscript is clearly written and relevant to ongoing EHDS implementation efforts. It offers a structured and practical roadmap for policymakers, Health Data Access Bodies (HDABs), ethics committees, and other stakeholders.

 The rationale for the paper is partly sufficient. The manuscript outlines the governance challenges posed by the EHDS and explains the need for clearer ethical guidance. However, the rationale would benefit from a more explicit articulation of the gaps in existing ethical frameworks and why this paper is the appropriate format for addressing them. The authors could strengthen the introduction by clarifying how current instruments fall short in operationalizing ethics for secondary health data use under the EHDS.

 The article partly references differing views and opinions. While it acknowledges multiple stakeholder groups (e.g. policymakers, HDABs, ethics committees, researchers, citizens, and healthcare professionals) it does not sufficiently highlight different or conflicting perspectives. Given the heterogeneity of national ethics procedures and varying interpretations of concepts such as public interest and proportionality, the manuscript would benefit from concrete examples illustrating these differences. This would enhance the reader’s understanding of why harmonization is challenging and necessary.

 The manuscript is written in accessible language. Technical concepts are explained clearly, and the plain language summary is particularly effective in communicating the core ideas to non‑expert audiences.

 The recommendations are clearly articulated and actionable. The structure makes them easy to follow and suitable for implementation by the research and policy community.  Including concrete examples of ethical dilemmas in secondary data use (e.g., AI model training, cross‑border linkage, rare disease registries) would also enhance practical applicability.

 Overall, this paper is a valuable and well‑structured contribution. 

 Thank you.

Where applicable, are recommendations and next steps explained clearly for others to follow? (Please consider whether others in the research community would be able to implement guidelines or recommendations and/or constructively engage in the debate)

Yes

Does the article adequately reference differing views and opinions?

Yes

Are all factual statements correct, and are statements and arguments made adequately supported by citations?

Yes

Is the rationale for the Open Letter provided in sufficient detail? (Please consider whether existing challenges in the field are outlined clearly and whether the purpose of the letter is explained)

Partly

Is the Open Letter written in accessible language? (Please consider whether all subject-specific terms, concepts and abbreviations are explained)

Yes

Reviewer Expertise:

Health data quality

I confirm that I have read this submission and believe that I have an appropriate level of expertise to confirm that it is of an acceptable scientific standard.
